# Derivation of Cinnamon Blocks Leukocyte Attachment by Interacting with Sialosides

**DOI:** 10.1371/journal.pone.0130389

**Published:** 2015-06-15

**Authors:** Wei-Ling Lin, Shih-Yun Guu, Chan-Chuan Tsai, Ekambaranellore Prakash, Mohan Viswaraman, Hsing-Bao Chen, Chuan-Fa Chang

**Affiliations:** 1 Department of Medical Laboratory Science and Biotechnology, College of Medicine, National Cheng Kung University, Tainan 70101, Taiwan; 2 Department of Biochemistry and Molecular Biology, College of Medicine, National Cheng Kung University, Tainan 70101, Taiwan; 3 Department of Pathology, Pingtung Christian Hospital, Pingtung 900, Taiwan; 4 Indus Biotech Private Limited, Kondhwa, Pune 411048, India; 5 Division of Colorectal Surgery, Department of Surgery, E-DA Hospital, Kaohsiung 82445, Taiwan; 6 Center of Infectious Disease and Signaling Research, College of Medicine, National Cheng Kung University, Tainan 70101, Taiwan; 7 Institute of Basic Medical Sciences, College of Medicine, National Cheng Kung University, 1 University Road, Tainan 70101, Taiwan; St. Jude Children's Research Hospital, UNITED STATES

## Abstract

Molecules derived from cinnamon have demonstrated diverse pharmacological activities against infectious pathogens, diabetes and inflammatory diseases. This study aims to evaluate the effect of the cinnamon-derived molecule IND02 on the adhesion of leukocytes to host cells. The anti-inflammatory ability of IND02, a pentameric procyanidin type A polyphenol polymer isolated from cinnamon alcohol extract, was examined. Pretreatment with IND02 significantly reduced the attachment of THP-1 cells or neutrophils to TNF-α-activated HUVECs or E-selectin/ICAM-1, respectively. IND02 also reduced the binding of E-, L- and P-selectins with sialosides. Furthermore, IND02 could agglutinate human red blood cells (RBC), and the agglutination could be disrupted by sialylated glycoprotein. Our findings demonstrate that IND02, a cinnamon-derived compound, can interact with sialosides and block the binding of selectins and leukocytes with sialic acids.

## Introduction

Cinnamon, a traditional Chinese herb, has been widely used as a spice in India, China, and many Asian countries. Cinnamon extracts, which contain several bioactive components, have also been studied for their anti-cancer [1–4], anti-infectious [5, 6], anti-diabetic [7–11] and anti-inflammatory responses [12–17]. For example, cinnamon extracts suppressed tumor progression and promoted tumor cell death through different signal pathways [1, 4]; crude cinnamon extracts showed significant anti-bacterial activities against foodborne pathogenic bacteria [5]; cinnamon extracts can also regulate glucose transporters, improve glucose uptake and fasting blood glucose, inhibit α-glucosidase activity and increase insulin sensitivity in diabetes or obesity [7, 8, 18–20]. Although the biological functions of cinnamon extracts have been widely studied, specific pharmaceutical applications and mechanisms of actions against various disease conditions remain uninvestigated.

Sialic acid, also called *N*-acetyl neuraminic acid (NeuAc), is a negatively charged, nine-carbon monosaccharide that is mostly located at the non-reducing end of glycoconjugates. Cell surface sialic acids have been reported to be involved in a variety of physiological and pathological functions, including cell-cell adhesion, recognition, migration, cancer progression and metastasis, as well as pathogen binding and infection. In addition, cell surface sialic acids are also ligands for selectins and participate in the rolling and recruitment of leukocytes during inflammation. Therefore, compounds that block protein-sialic acid binding can prevent infection by pathogens; the tethering, rolling, and adhesion of leukocyte to the extravascular sites; and the subsequent chemokine releasing and inflammation [21–25].

Using chromatography, we purified polyphenol-rich, alcohol-soluble compounds from pulverized cinnamon (*Cinnamomum zylanicum*) bark. After HPLC separation and NMR characterization, the major component of the purified polyphenol is composed of a pentameric procyanidin type A polyphenol polymer called IND02 [15]. We first examined the effects of IND02 on the adhesion of THP-1 cells to HUVECs and the attachment of neutrophils to E-selectin/ICAM-1. The glycan binding preferences of recombinant E-, L- and P-selectins were then characterized. We also analyzed the inhibition abilities of IND02 in selectin-glycan interactions. Finally, we tried to determine the possible anti-inflammatory mechanisms of IND02. Based on our findings, IND02 can interact with sialosides and reduce the interactions of leukocytes with cells.

## Materials and Methods

### Purification and characterization of IND02

The cinnamon-derived compound, IND02, was provided by Indus Biotech Private Limited, Pune, India and characterized as described (PCT Publication No: 011/018793A1, WO2011018793 A1) [15, 16]. IND02 is a standardized alcohol extract of *Cinnamomum zylanicum* bark with pentameric type-A procyanidine flavonoid (S1 Fig). The solutions of IND02 were freshly prepared by dissolving in methanol or double distilled water.

### Cells

Human leukemia monocytic THP-1 cells were purchased from Bioresource Collection and Research Center (BCRC, Hsinchu, Taiwan) and cultured in RPMI-1640 medium (GIBCO BRL, Grand Island, USA). Human umbilical vein endothelial cells (HUVECs) were purchased from Invitrogen (NY, USA) and cultured in Medium 199 (GIBCO BRL, Grand Island, USA). Cells were cultured by the recommended condition. HUVECs from passage 2 to 4 were used in the experiments.

### Monocytic cells adhesion assay

HUVECs were grown to confluence on the flow chamber slide (μ-Slide I^*0*.*8*^ Luer, ibidi, Martinsried, Germany) and activated by tumor necrosis factor (TNF)-α (10 ng/ml) for 16–18 h. Before the assay, THP-1 monocytic cells (5x10^*5*^/ml) were preincubated with or without IND02 (200 μg/mL, final concentration) for 30 minutes at 37°C. After washing HUVECs by serum-free medium, THP-1 cells were perfused in the flow chamber at a constant flow rate of 0.5 dynes/cm^*2*^. The number of adherent THP-1 cells was determined after 20 minutes of perfusion. In these experiments, the shear flow was generated using the perfusion loop system and an air pressure pump (ibidi, Martinsried, Germany), and images were recorded every 10 seconds. The number of adherent cells was calculated in 10–15 random fields in a single experiment and, the statistical test were calculated from 3 independent experiments.

### Isolation of human neutrophils

As previously described [26], whole blood was collected into tubes containing heparin. After RBC sedimentation by 3% dextran T-100 (Sigma-Aldrich, USA), leukocyte-rich plasma was centrifuged at 1000 rpm for 10 minutes to pellet cells. Cells were then resuspended by saline, and Ficoll-Hypaque (Pharmacia, USA) solution was layered beneath the cell suspension. After centrifuging at 1400 rpm for 40 minutes, supernatant was removed to leave pellet consisting of neutrophils and residual RBC. After removing RBC by hypotonic lysis, neutrophils were resuspended in ice-cold PBS/glucose. Cells were used within 2 hours. The obtaining of human cells was approved by the institutional review board of National Cheng Kung University Hospital, Tainan, Taiwan.

### Neutrophils adhesion assay

For adhesion of neutrophils to immobilized E-selectin in combine with ICAM-1 under flow, a laminar flow chamber slide (μ-Slide I^*0*.*8*^ Luer, ibidi, Martinsried, Germany) was filled with protein G (25 μg/mL; Invitrogen, USA) overnight. After washing with phosphate-buffered saline, the slide was filled with E-selectin (5 μg/mL; R&D system, USA) and ICAM-1 (5 μg/mL; R&D system, USA) for 2 hours and blocked with 1% bovine serum albumin (Sigma-Aldrich, USA) for 1 hour. Freshly isolated human neutrophils (5x10^*5*^ cells) were preincubated without or with IND02 at RT for 1 hour, followed by perfusion at a constant flow rate of 1 dynes/cm^*2*^. The number of adherent cells (stationary over 5 seconds) was determined after 3 minutes of perfusion. The shear flow was generated by using the perfusion loop system and an air pressure pump (ibidi, Martinsried, Germany), and the images were captured by the microscope. The number of adherent cells was calculated in 10–15 random fields in a single experiment and the statistical test was calculated from 3 experiments.

### Carbohydrate binding specificities of selectins (AlphaScreen assay)

AlphaScreen assays were performed on a PerkinElmer Envision instrument. Streptavidin-coated donor beads, protein A-conjugated acceptor beads and ProxiPlate-384 assay plates were purchased from PerkinElmer Life Sciences, Inc. (Boston, MA, U.S.A.). Biotinylated polyacrylamide-based sugar polymers were purchased from GlycoTech (Gaithersburg, MD, U.S.A.). Anti-human IgG antibody was purchased from Acris (BM478, Herford, Germany). Rabbit anti-mouse IgG was purchased from Zymed, Inc. (South San Francisco, CA, U.S.A.). Recombinant human selectin Fc chimera proteins (including E-, L- and P-selectins) were purchased from R&D Systems (Minneapolis, MN, U.S.A.). All of the procedures and incubations were performed in the dark. The buffer used in selectin assay was 25 mM Tris buffer, pH = 7.0, 1.0 mM CaCl_*2*_, 0.1% BSA). Donor beads (20 ng/well) and biotin-PAA-sugars (20 ng/well) mixed with selectins (5 ng/well) were incubated at ambient temperature for 1 h (total 15 μL). The mixture of acceptor beads (20 ng/well), mouse anti-human IgG antibody (50 ng/well) and rabbit anti-mouse IgG antibody (25 ng/well) was added to the reaction to a final volume of 25 μL. After 2 h of incubation, the binding signals were obtained using a PerkinElmer Envision instrument and the AlphaScreen program. The optimized concentrations of selectins were measured and used for binding assays. The results were depicted using units of relative intensities.

### Amplification and purification of influenza A virus

The human influenza A virus (seasonal H1N1 strains, A/Taiwan/211/2009) used in this study was collected from the Virology Laboratory of National Cheng Kung University Medical Center. The virus was amplified in MDCK cells and purified using a sucrose gradient. The purified virus was inactivated by treating with 0.01% Merthiolate (final concentration). The protein concentrations of the purified virus were determined by a protein assay (Bio-Rad Laboratories, CA, USA).

### RBC agglutination and inhibition assays

Two-fold serially diluted influenza A virus in row A and B (column 1 to 11, 50 μg/mL to 50 ng/mL) and two-fold serially diluted IND02 in row C and D (column 1 to 11, 50 μg/mL to 50 ng/mL) were prepared in the microtiter plate. Washed human RBCs from blood bank of National Cheng Kung University Hospital (5% v/v) were added to the wells and incubated at 37°C for 1 h. The agglutination titers of influenza A virus and IND02 were 1:16 (3.125 μg/mL) and 1:64 (0.78125 μg/mL), respectively.

Two-fold serially diluted fetuin (column 1 to 11, 50 μg/mL to 50 ng/mL) were prepared. Influenza A virus (row A and B, 10 μg/mL), IND02 (row C and D, 10 μg/mL), or PBS (row E and F) were added and incubated with fetuin at 37°C for 0.5 h. Washed RBCs (5%) were then added to the reaction and incubated at 37°C for 1 h.

### Statistical analysis

**Fig 1:**
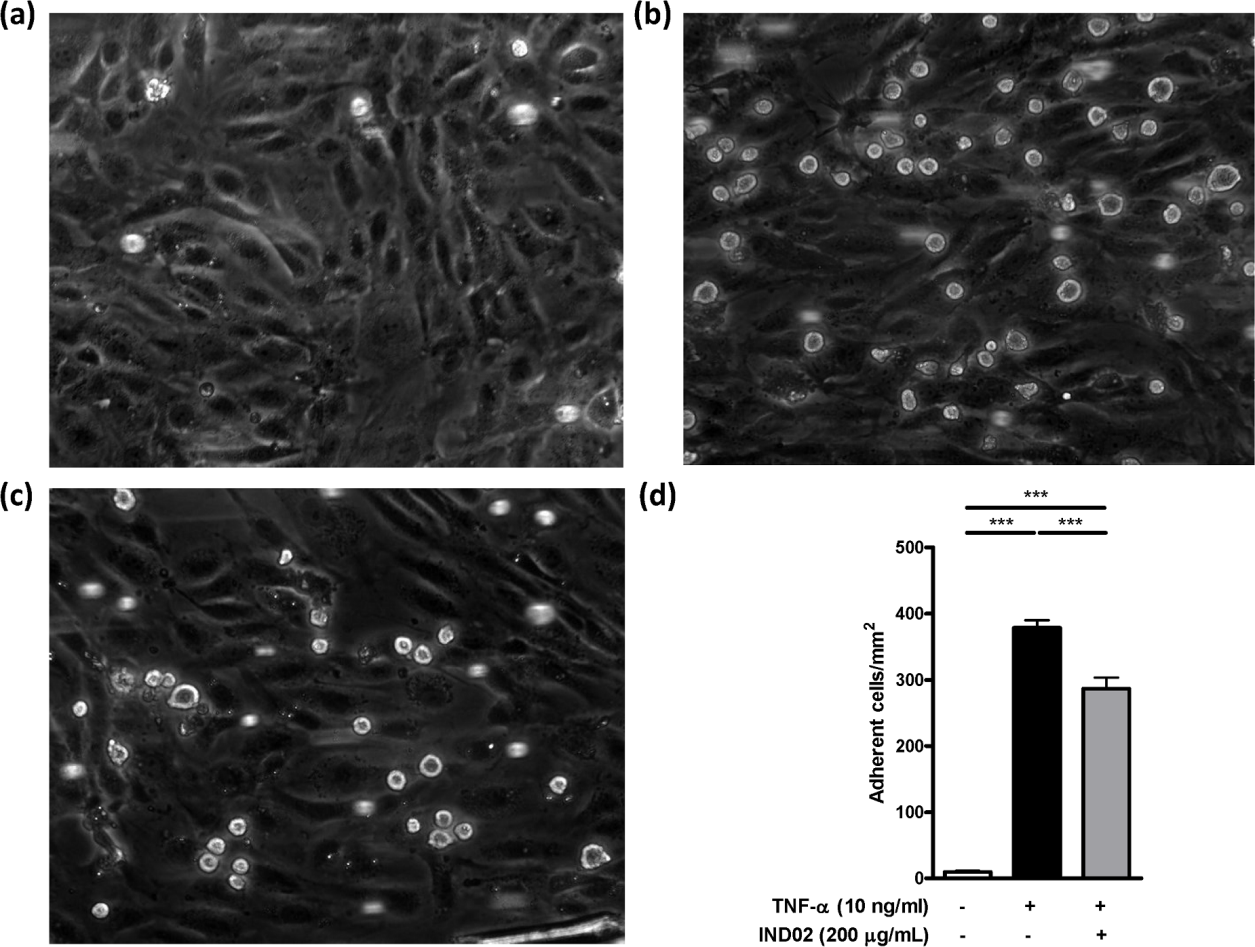
Monocytes adhesion assay. (a) THP-1 monocytic cells were preincubated with PBS for 0.5 h at 37°C followed by perfusion into a flow chamber. The rolling (attachment) of THP-1 with HUVECs (without TNF-α activation) were recorded and captured. (b, c) THP-1 monocytic cells were preincubated with PBS (b) or IND02 (200 μg/mL) (c) for 0.5 h at 37°C followed by perfusion into flow chamber. The rolling (attachment) of THP-1 with TNF-α activated HUVECs were recorded and captured. (d) Control (without TNF-α activation): 9.86±1.81 cells/mm^2^; Control (TNF-α activated HUVEC): 378.5±11.5 cells/mm^2^; IND02 (200 μg/mL): 286.3±17.3 cells/mm^2^. The number of adherent cells was calculated in 10–15 random fields in a single experiment, and the statistical test were calculated based on 3–5 experiments using one-way ANOVA followed by post hoc test. The movie of (a)-(c) were showed in S1–S3 Movies. IND02 significantly reduced THP-1 attachment and rolling on TNF-α activated HUVECs. (***: *P* < 0.001).

**Fig 2:**
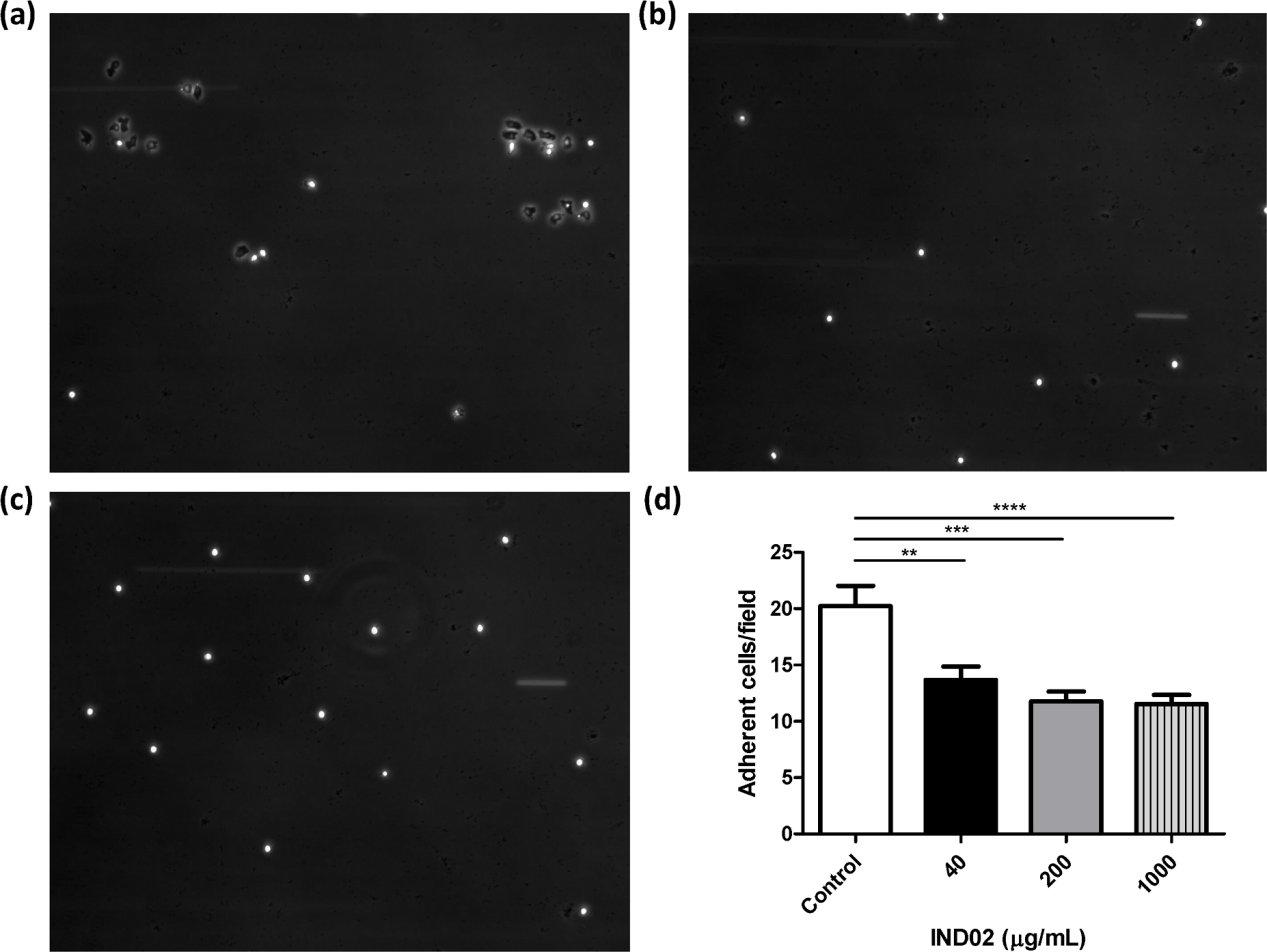
Neutrophils attachment assay. Neutrophils were preincubated with (a) PBS, (b) IND02 (1000 μg/mL), (c) IND02 (40 μg/mL), for 0.5 h at 37°C followed by perfusion into a flow chamber. The attachment of neutrophils with E-selectin/ICAM-1 coated slides were recorded and captured. (d) Control: 20.2±6.8 cells/field; IND02 (40 μg/mL): 13.7±3.6 cells/field; IND02 (200 μg/mL): 11.8±2.5 cells/field; IND02 (1000 μg/mL): 10.9±2.8 cells/field. The number of attached cells was calculated in 10–15 random fields in a single experiment, and the statistical test were calculated based on 3–5 experiments using one-way ANOVA followed by post hoc test. IND02 significantly reduced neutrophils attachment and rolling on E-selectin/ICAM-1 coated slides. (**: *P* < 0.01; ***: *P* < 0.001; ****: *P* < 0.0001).

All of the results were obtained from at least three independent experiments. The data were analyzed using Student’s t test (two-tailed) or one-way ANOVA followed by post hoc test (Figs 1d and 2d) on GraphPad Prism 5 software. P values of less than 0.05 were considered to be statistically significant. (*: *P* < 0.05; **: *P* < 0.01; ***: *P* < 0.001)

## Results

### IND02 reduced leukocyte attachment

Cinnamon extracts have been shown to exhibit anti-inflammatory activity in several experimental models [12–14]. The primary response of the innate immune system to inflammation is the recruitment of leukocytes. To investigate whether IND02 affects this first step of inflammation (leukocyte attachment), we analyzed the adhesion of THP-1 cells with TNF-α activated HUVECs under shear flow with or without IND02 pretreatment. IND02 significantly reduced the adhesion of THP-1 cells to TNF-α-activated HUVECs when compared with untreated group. (Fig 1 and S1–S3 Movies). In addition, the effects of IND02 on neutrophil attachment to E-selectin and ICAM-1 coated on slides were also evaluated. We observed that neutrophils were able to spread in the absence of IND02 (Fig 2a); however, the shape of cell retained round up in the pretreatment of IND02 (Fig 2b). Moreover, the number of adherent neutrophils to E-selectin/ICAM-1-coated surface was significantly reduced by the treatment of IND02 (Fig 2c). To confirm the viability of the attached neutrophils, the slides were stained with trypan blue. The viability of the attached neutrophils was higher than 95% (data not shown). These results suggested that IND02 may inhibit inflammatory responses by blocking leukocyte adhesion.

### IND02 blocked the binding of selectins with sialosides

**Fig 3:**
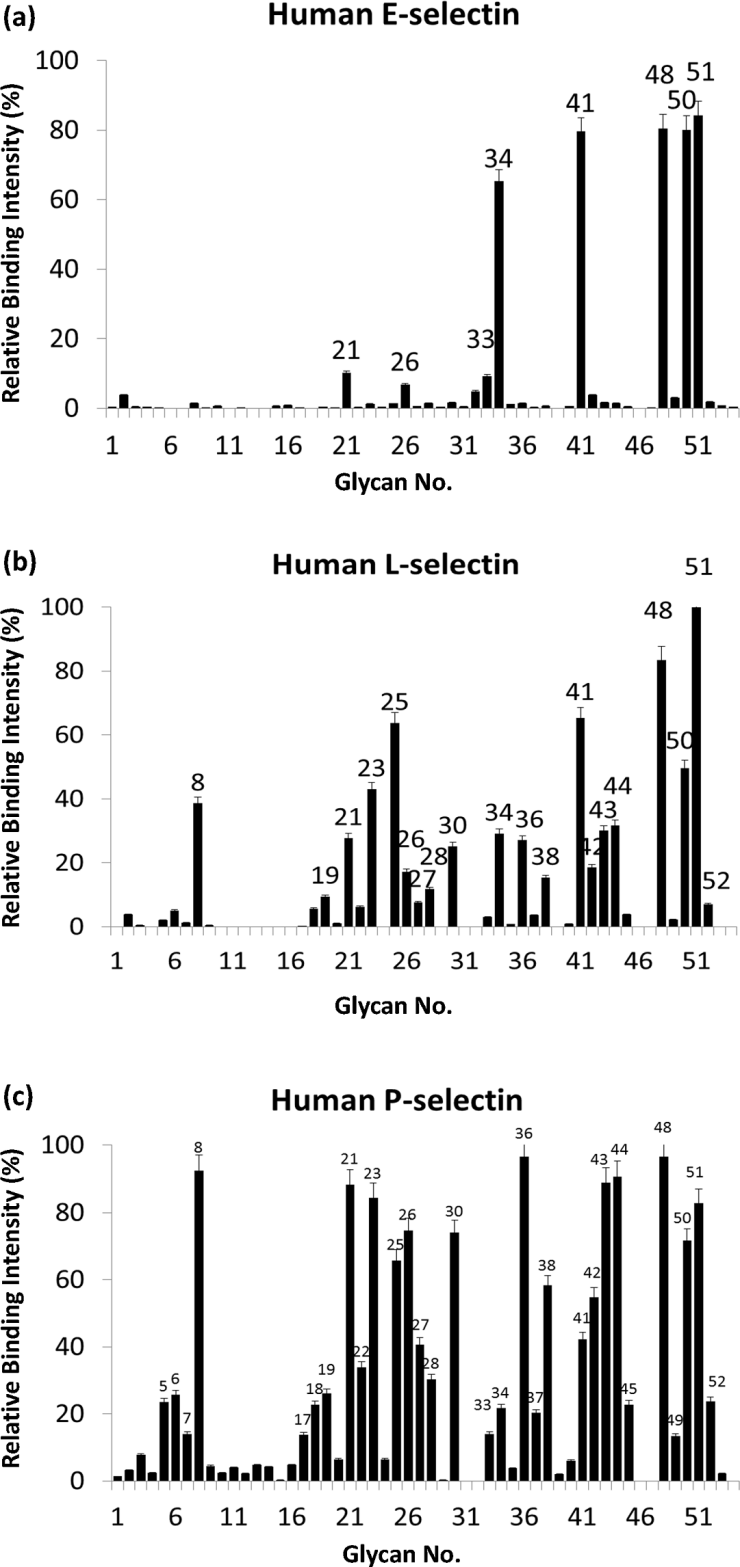
Carbohydrate binding specificities of E-, L- and P-selectin. Donor beads and biotin-PAA-sugars mixed with selectins were incubated at ambient temperature for 1 h (total 15 μL). The mixture of acceptor beads, mouse anti-human IgG antibody and rabbit anti-mouse IgG antibody was added to the reaction to a final volume of 25 μL. After 2 h of incubation, the binding signals were obtained using a PerkinElmer Envision instrument and the AlphaScreen program. The results were are indicated by relative intensities of the highest fluorescence signal (*y-axis*). The sugar identities are designated by numbers (*x-axis*) and shown in detail in S1 Table. Glycans which showed more than 30% relative intensity are indicated as number on each figure.

**Fig 4:**
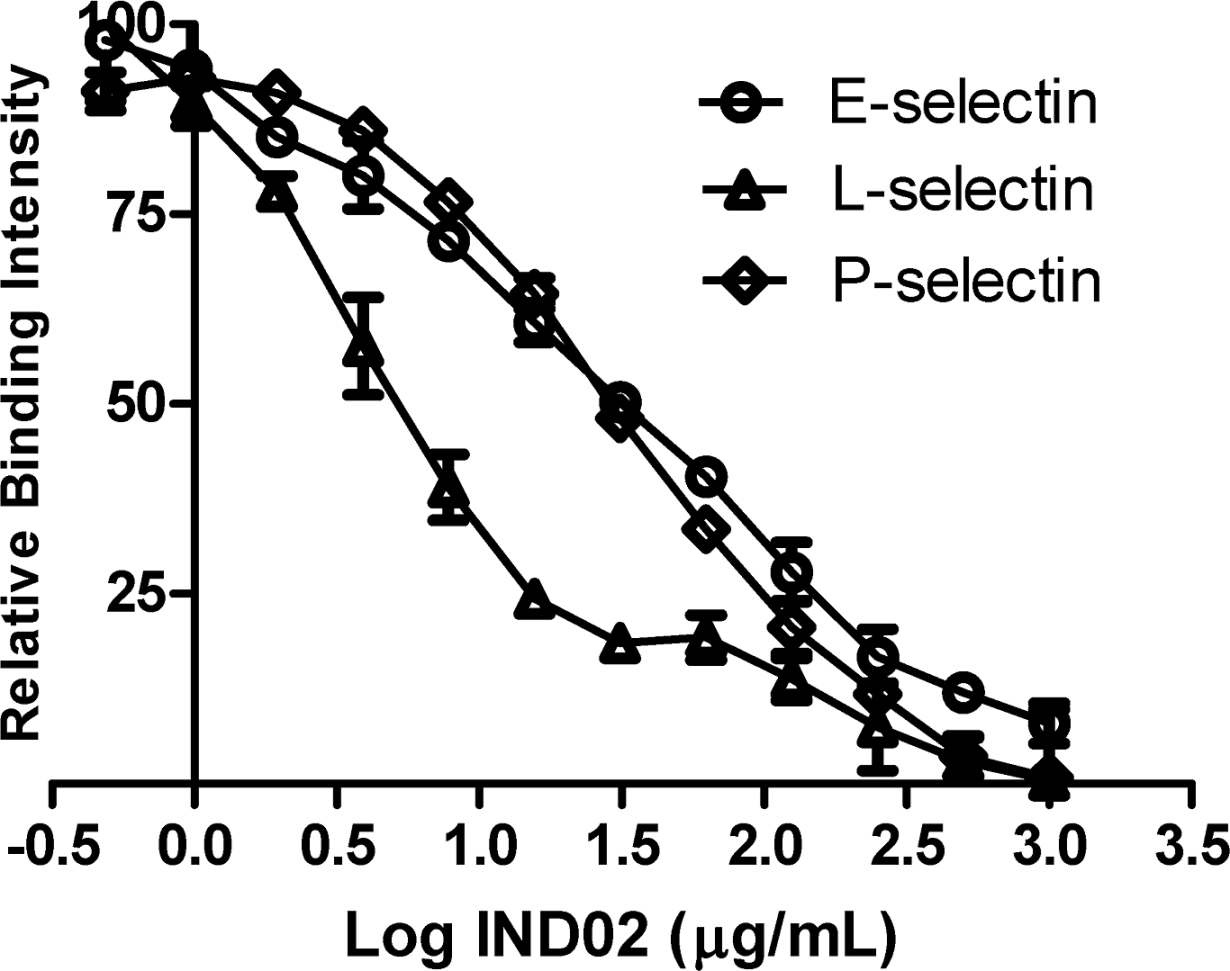
IND02 inhibited the binding of E-, L- and P-selectin to sialyl Le^x^. E-, L- and P-selectin were incubated with biotin-PAA-sialyl Le^x^ for 1 h in a 384 microtiter plate. Serially diluted IND02 (from 1000 μg/mL to 0.5 μg /mL) was added to each well, incubated at room temperature for 2 h and detected using a reader. Based on the relative binding intensity, the IC_50_ values of IND02 for E-, L- and P-selectin were 31.6, 5.0 and 28.2 μg/mL, respectively. The results were the average of six independent assays.

Because the recruitment of neutrophils/leukocytes to inflamed tissue is mediated by selectin-sialoside interactions, human selectins were subjected to carbohydrate binding and IND02 inhibition assays to explore the molecular mechanism of IND02 in leukocyte recruitment. The substrate specificities of recombinant human E-, L- and P-selectins (human Fc chimera, purchased from R&D) were profiled primarily by solution glycan microarray [27]. The PAA-glycans used in this binding assay are listed in S1 Table. E-selectin preferentially bound to Le^b^, sialyl-Le^a^, sialyl Le^x^, and 3’sulfated Le^a^/Le^x^ (Fig 3a, relative intensity>30%). L-selectin interacted with sulfated glycans, NeuGc, 6’sialyl lactose, NeuAcα2-3Galβ1-4GlcNAc, NeuAcα2-3Galβ1-3GalNAc, Le^b^, sialyl-Le^a^ and sialyl Le^x^ (Fig 3b, relative intensity>30%). P-selectin recognized several glycans including sulfated glycans, NeuGc, NeuAcα2-6GalNAc, NeuGcα2-6GalNAc, NeuAcα2-3Gal, NeuAcα2-3GalNAc, 6’sialyl lactose, 3’6’disialyl-GalNAc, Galα1-4Galβ1-4Glc, NeuAcα2-3Galβ1-4GlcNAc, NeuAcα2-3Galβ1-3GalNAc, Le^b^, sialyl-Le^a^ and sialyl Le^x^ (Fig 3c, relative intensity>30%). Among the common ligands for the three recombinant human selectins, the common ligand of selectins, sialyl Le^x^, was used to analyze the IC_50_ of IND02 [28]. Serially diluted IND02 (from 0.5 μg/mL to 1000 μg /mL) were preincubated with biotin-PAA-sialyl Le^x^ and then interacted with selectins. The results showed that the IC_50_ values of IND02 for E-, L- and P-selectin were 31.6, 5.0 and 28.2 μg/mL, respectively (Fig 4).

### IND02 agglutinated human red blood cells by interacting with sialosides

**Fig 5:**
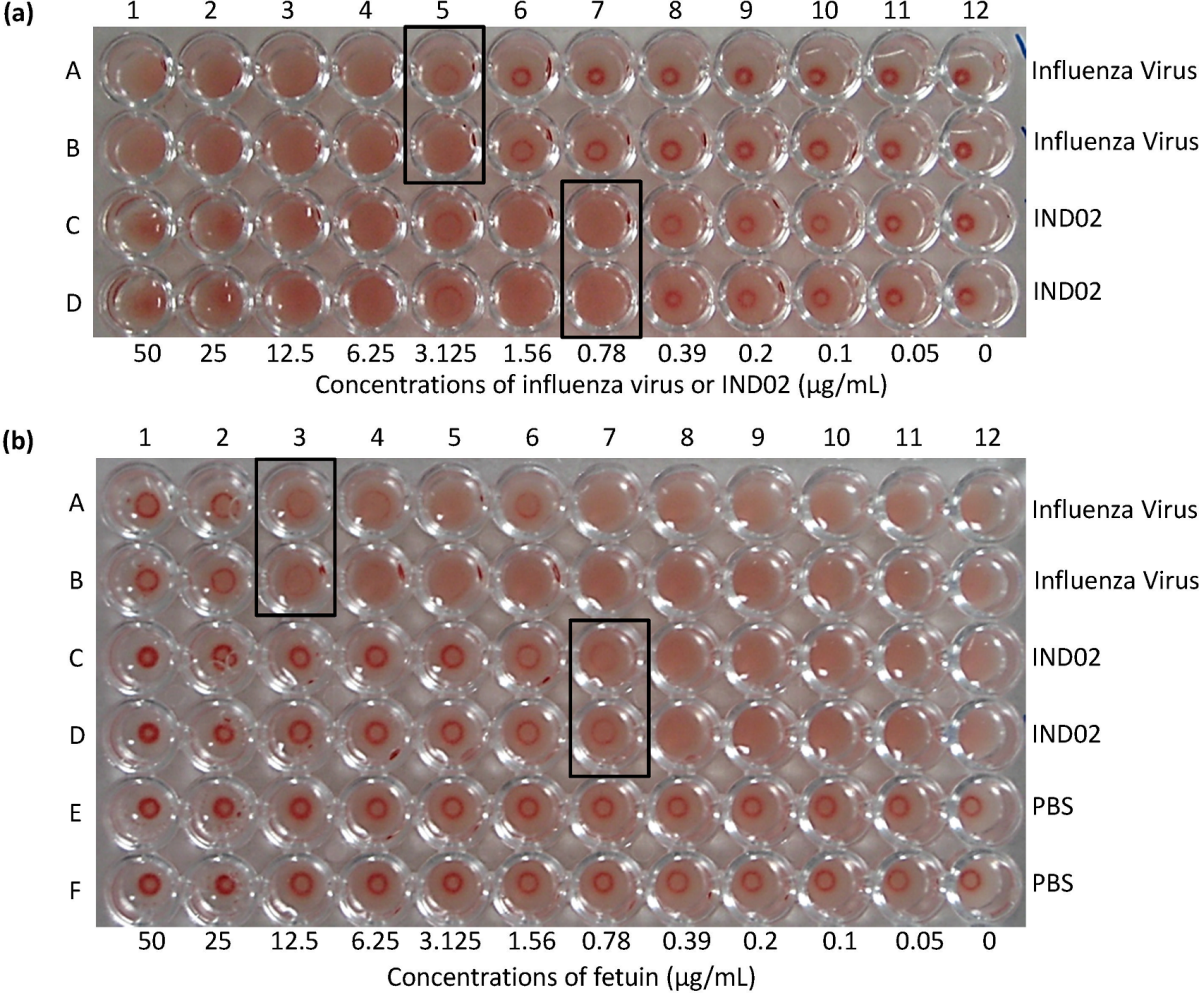
(a) Agglutination assays of the influenza virus and IND02. Two-fold serially diluted influenza A virus in row A and B (column 1 to 11, 50 μg/mL to 50 ng/mL) and two-fold serially diluted IND02 in row C and D (column 1 to 11, 50 μg/mL to 50 ng/mL) were prepared in the microtiter plate. Washed human RBCs (5% v/v) were added to the wells and incubated at 37°C for 1 h. The agglutination titers of influenza A virus and IND02 were 1:16 (3.125 μg/mL) and 1:64 (0.78125 μg/mL), respectively. (b) Inhibition assay. Two-fold serially diluted fetuin (column 1 to 11, 50 μg/mL to 0 ng/mL) were prepared. Influenza A virus (as control, row A and B, 10 μg/mL), IND02 (row C and D, 10 μg/mL), or PBS (row E and F) were added and incubated with fetuin at 37°C for 0.5 h. Washed human RBCs (5%) were then added to the reaction and incubated at 37°C for 1 h. The results showed that the lowest fetuin concentration that block human RBC agglutinations for the influenza virus and IND02 were 12.5 μg/mL and 0.78125 μg/mL, respectively.

Influenza virus can agglutinate animal RBCs through the binding of hemagglutinin, one of the major proteins on influenza viral envelop, with cell surface α2–3 or α2–6 linked sialic acids [29]. The levels of influenza virus in sample can be quantified by hemagglutination test (HA test). The ability of compounds or proteins that prevent hemagglutination can be determined by hemagglutination inhibition test (HI test) [30]. Since we found that IND02 may interact with sialosides and reduce the binding of selectins to carbohydrate ligands, we tried to assess the inhibition capacity of IND02 using HI test. Unexpectedly, IND02 agglutinated human RBC independently of influenza virus addition, and the HA titer of influenza A virus and IND02 were 1:16 (3.125 μg/mL) and 1:64 (0.78125 μg/mL), respectively (Fig 5a). To investigate whether sialosides were involved in IND02 induced human RBC agglutination, fetuin, a highly sialylated glycoprotein, was used to disrupt these interactions [31]. As shown in Fig 5b, the addition of fetuin significantly reduced the influenza A virus and IND02-induced human RBC agglutination. These findings suggested that IND02-induced human RBC agglutination was due to interactions of IND02 with cell surface sialosides.

## Discussion

Cell migration is important in innate and adaptive immune responses and the recruitment of leukocytes to the sites of acute or chronic inflammation [21, 32, 33]. Inadequate congregation of leukocytes may reduce inflammatory responses and initiate pathological processes of inflammation [32, 33]. Cell surface proteins, especially members of the integrin family and E-selectins of endothelial cells and L-selectins of leukocytes, are known to participate in cell-cell adhesion and trafficking [32, 34–36]. In addition, P-selectin expressed on platelets is also involved in these processes [37–39]. Selectins are single-chain transmembrane glycoproteins bearing C-type lectins that contain calcium-dependent, carbohydrate recognition domains (CRDs). The CRDs of selectin recognize sialylated glycans and control the interactions between leukocytes and endothelial cells. The recruitment of leukocytes is always accompanied by the activation and expression of E-, P- and/or L-selectin ligands. The majority of the ligands for selectins are highly glycosylated, especially with sialyl Le^x^ glycans [40]. Although the affinity of selectins to sialyl Le^x^ is weak (with a dissociation constant in μM range), multivalent effects can compensate for the weak interactions [41–43].

Several selectin-directed anti-inflammatory compounds/strategies have been developed and evaluated in preclinical trails, including carbohydrate-based inhibitors, non-carbohydrate molecules, peptides, antibodies and nucleotide inhibitors [21, 44]. For example, P-selectin inhibitors reduce platelet aggregation, prolong heart allograft survival, decrease vein injury from thrombosis, and reduce acute pancreatitis in murine model [45–48]. Non-anticoagulant (NAC) heparin, a well-known selectin inhibitor, reduces tumor metastasis in selectin knockout mice [49, 50]. The pan-selectin antagonist Bimosiamose, a mannose-based dimer, could reduce airway inflammation [51]. Cinnamon extracts have also been reported to show anti-inflammatory responses [12–17]. However, the real roles of cinnamon extracts in reducing inflammatory responses are still unclear. In this study, we found that IND02 reduced the attachment of THP-1 to TNF-α activated HUVECs under shear flow. IND02 also attenuated the attachment of human neutrophils to E-selectin/ICAM-1-coated surface. E-selectin plays a critical role in mediating leukocyte rolling by interacting with sialic acids expressed on leukocyte surface. Therefore, E-selectin is a necessary adhesion molecule involved in the transition of leukocyte rolling to adhesion. Based on these findings, it is suggested that cinnamon extracts can interfere with selectin-sialosides interaction directly and resulted in a reduced leukocytes attachment.

While leukocytes stay in resting, the adhesion molecules are homogeneously expressed on the leukocyte surface. When leukocytes are rolling over the activated endothelium, the adhesion molecules on the surface of leukocytes are going to be redistributed via signals induced by endothelial adhesion molecules, such as E-selectin. Redistribution of adhesion molecules on leukocytes can increase the binding avidity to their counter-act receptors and enhance leukocyte adhesion. In this study, neutrophils can adhere and spread out under the flow condition; however, under the treatment of IND02, the adherent neutrophils were easily detached. The morphology of neutrophils was also very different between control and IND02 treated cells (Fig 2a–2c). It is suggested that the distribution of adhesion molecules or arrangement of cytoskeleton is affected by IND02. Although THP-1 and neutrophils both belong to myeloid cell linage, the characteristics of these two types of cells are different. After neutrophils were arrested, the cells spread to become firm adhesion. The transit from round up to spread out was apparent. However, we did not observe such morphology differences before and after the treatment of IND02 in THP-1 cells. It is possible that cells would have different behavior when adhesion to E-selectin/ICAM-1-coated surface or activated endothelial cells. Further studies are needed to dissect the detailed mechanisms, to investigate whether IND02 interfere with E-selectin-induced signaling, and to study whether IND02 interfere with the expression level of adhesion molecules.

To investigate the inhibitory mechanism of IND02, we analyzed the carbohydrate binding specificities of recombinant selectins. The recombinant selectins not only showed interactions with sialosides, especially to sialyl Le^x^, but also bound to some glycans without sialic acid modification [52]. Hence, PAA-sialyl Le^x^ was subjected for the subsequent assays. It is meaningful to use sialyl Le^x^ in the inhibition assays because sialyl Le^x^ is the natural ligand for selectins. The results showed that the binding of selectins with sialyl Le^x^ dropped significantly after IND02 treatments. These findings suggest that selectin-sialic acid interactions may be one of the targets for IND02.

To further elucidate the possible inhibitory mechanisms for IND02, we performed HA test, HI test, and glycoprotein blocking assay for IND02 [53]. Surprisingly, IND02 agglutinated human RBC in a dose dependent manner. The ability of RBC agglutination for IND02 may lead to some possible side effects. IND02 may cause aggregation and obstruct small vessel in some special condition. However, it is known that influenza virus can agglutinate human RBC in 96-well assays, but will not agglutinate RBC in blood vessel. To verify whether the IND02-induced agglutination was sialic acid dependent, a well-known inhibitor for RBC agglutination, fetuin, was subjected to a blocking assay. We found that IND02-induced human RBC agglutination was significantly reduced by fetuin treatment. Taken together, this evidence suggested that IND02 exhibits anti-inflammatory activity by interacting with cell surface sialosides to reduce leukocyte attachment. Injection of IND02 into the vein or the site of inflammation may reduce leukocyte recruitment and decrease the subsequent leukocyte infiltration.

In conclusion, we show for the first time that IND02, a cinnamon-derived type A procyanidin molecule, reduces inflammatory responses by interaction with sialosides and blocking leukocyte adhesion to interacting sialosides. These findings not only confirm the reported anti-inflammatory abilities of IND02, but also dissect the inhibition mechanisms of IND02. Further studies are in progress to explore novel biological functions and anti-inflammatory/anti-infection mechanisms for IND02.

## Supporting Information

S1 Fig HPLC analysis and structure of IND-02. (a) HPLC absorbance chromatogram (at 280 nm) of alcohol-soluble compounds from pulverized cinnamon (*Cinnamomum zylanicum*) bark. 4% of the eluted material is procyanidin trimer and 92% of the eluted material are pentamer polyphenols (IND-02). Unknown material (peak 1, 2, 4 and 5) represents 4.1% of the total UV trace. (b) Structure of the pentameric procyanidin A (IND-02).
(TIF)

S1 Movie Negative control. THP-1 monocytic cells were preincubated with PBS for 0.5 h at 37°C followed by perfusion into a flow chamber. The rolling (attachment) of THP-1 with HUVECs were recorded.
(MP4)

S2 Movie Positive control. THP-1 monocytic cells were preincubated with PBS for 0.5 h at 37°C followed by perfusion into a flow chamber. The rolling (attachment) of THP-1 with TNF-α activated HUVECs were recorded.
(MP4)

S3 Movie IND02 treatment. THP-1 monocytic cells were preincubated with IND-02 (200 μg/mL) for 0.5 h at 37°C followed by perfusion into a flow chamber. The rolling (attachment) of THP-1 with TNF-α activated HUVECs were recorded.
(MP4)

S1 Table Glycan list for selectin binding assay. (DOCX)
