# Supplementary figures and images for: Derivation of Cinnamon Blocks Leukocyte Attachment by Interacting with Sialosides
Source: PLoS One. 2015 Jun 15;10(6):e0130389. doi: 10.1371/journal.pone.0130389 (PMC4468131; doi:10.1371/journal.pone.0130389)

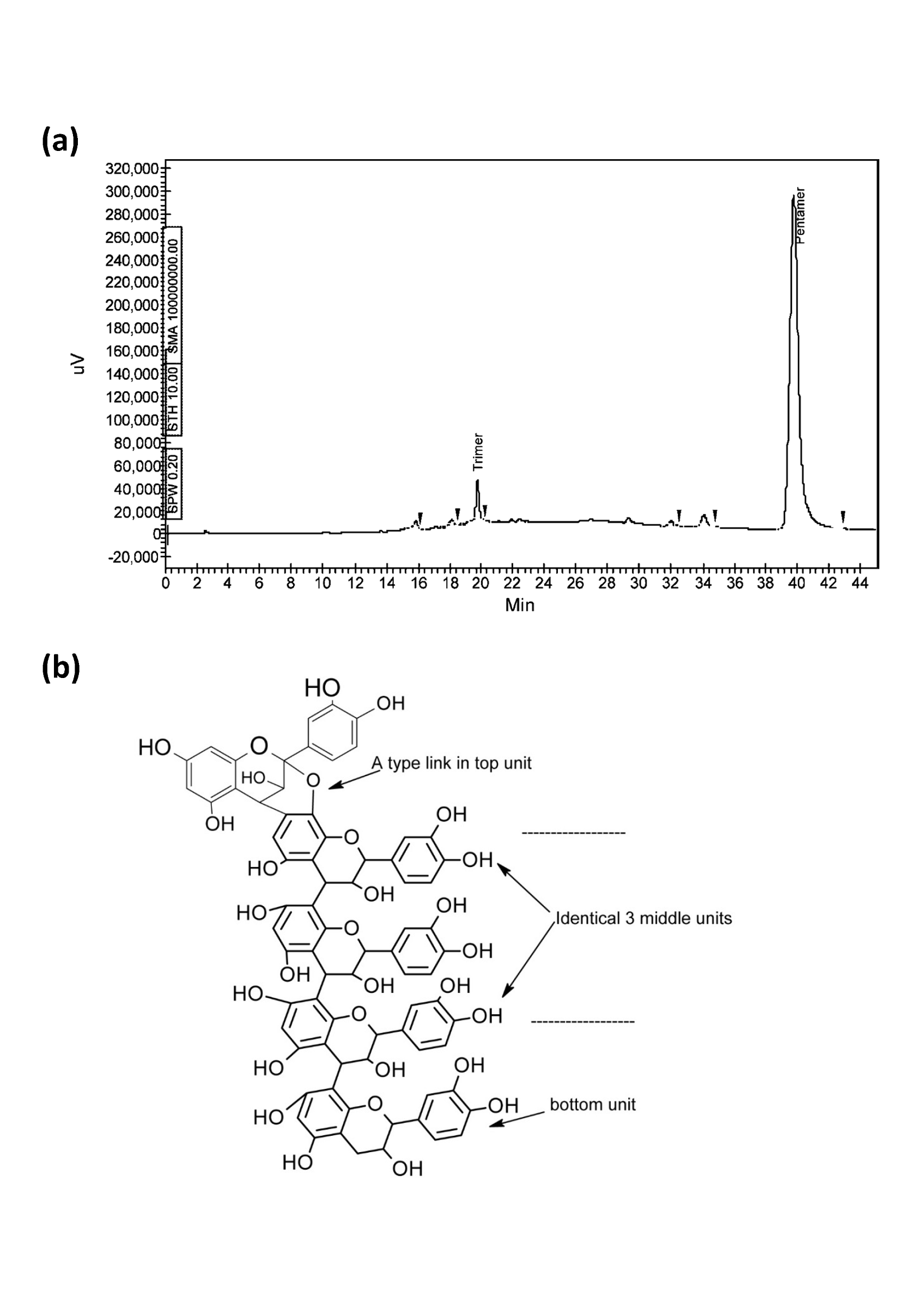

Supplement: S1 Fig — (a) HPLC absorbance chromatogram (at 280 nm) of alcohol-soluble compounds from pulverized cinnamon (Cinnamomum zylanicum) bark. 4% of the eluted material is procyanidin trimer and 92% of the eluted material are pentamer polyphenols (IND-02). Unknown material (peak 1, 2, 4 and 5) represents 4.1% of the total UV trace. (b) Structure of the pentameric procyanidin A (IND-02). (TIF) [file pone.0130389.s001.tif]

**S1 Table. Glycan list for selectin binding assay**


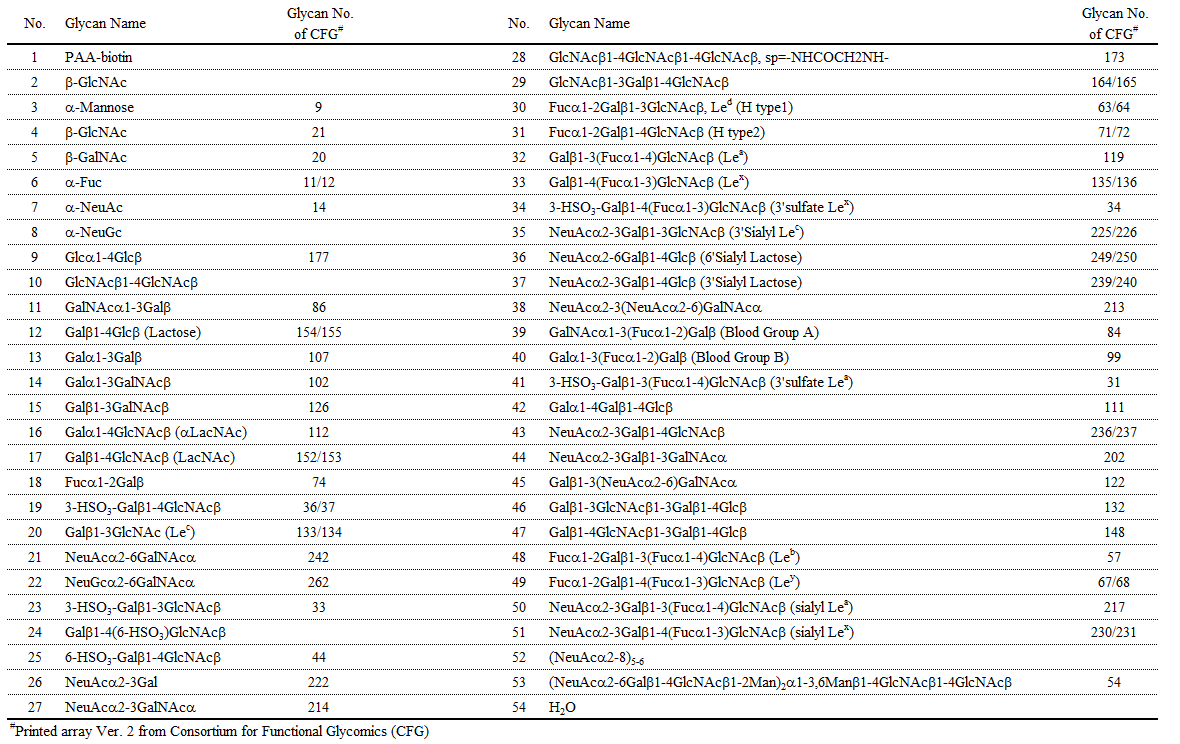

Supplement: S1 Table — (DOCX) [file pone.0130389.s005.docx]
